# Nanomaterials for Chemical Engineering (3rd Edition)

**DOI:** 10.3390/nano16171094

**Published:** 2026-09-01

**Authors:** Meiwen Cao

**Affiliations:** State Key Laboratory of Heavy Oil Processing and Department of Biological and Energy Chemical Engineering, College of Chemistry and Chemical Engineering, China University of Petroleum (East China), Qingdao 266580, China; mwcao@upc.edu.cn

## 1. Introduction

Over the past decades, nanomaterials have emerged as a transformative force across a broad spectrum of technology- and industry-driven sectors, offering unprecedented opportunities to address long-standing challenges in chemical engineering. The ability to manipulate matter at the nanoscale enables the design of materials with extraordinary properties—ranging from exceptional catalytic activity and selective adsorption to tunable optical, electrical, and mechanical behaviors. Nevertheless, the precise control over morphology, size, porosity, conductivity, and chemical activity remains a formidable task, requiring synergistic advances in synthesis protocols, characterization techniques, and computational modeling. The main applications of nanomaterials in chemical engineering span catalysts, functional coatings, adsorbents, sensors, drug-delivery vehicles, and beyond, all of which represent fascinating yet demanding research frontiers.

Following the success of the two previous editions of this Special Issue, “Nanomaterials for Chemical Engineering” [1,2], which collectively gathered 34 high-quality contributions, this third edition continues to serve as a vibrant platform for disseminating cutting-edge research on the synthesis, functionalization, and application of nanomaterials in chemical engineering contexts. The present collection comprises eleven original research articles, communications, and a review, reflecting the diversity and interdisciplinary nature of the field. The contributions span metal-matrix nanocomposites, surface engineering, fluidization technology, bioceramic sealers, advanced functional coatings, cultural heritage conservation, machine learning-assisted materials design, and bicontinuous soft materials. Below, I provide a brief overview of each contribution to guide the readers through the rich content of this Special Issue.

## 2. An Overview of Published Articles

The first contribution by the authors Seyit Çağlar and Cengiz Temiz (Contribution 1) reports a hybrid processing route combining melt-spinning, mechanical alloying, and sintering to fabricate Al-5Cu-0.3Sc matrix composites reinforced with 0–20 wt.% B_4_C. Their detailed microstructural analysis reveals a dual-strengthening mechanism, that is, precipitation strengthening from Al_2_Cu/Al_3_Sc intermetallics coupled with particle strengthening from B_4_C, which elevates hardness by approximately 319% and improves wear resistance by nearly 60-fold. Notably, the work also highlights a critical performance trade-off: the enhanced mechanical and tribological properties are accompanied by a significant increase in corrosion rate driven by microgalvanic coupling, offering valuable design insights for lightweight structural applications.

Huixing Zhang and co-workers (Contribution 2) address the ubiquitous problem of surface fogging on glass and quartz through a programmed fast plasma treatment performed in ambient air. In contrast to conventional thermal annealing, their method achieves a 0° water contact angle within seconds without sacrificing optical transmission, and it readily scales to 30 × 30 cm^2^ substrates. This rapid, energy-efficient, and scalable approach holds substantial promise for automotive, medical, and display technologies where optical clarity is paramount.

In Contribution 3, Syed Sadiq Ali and colleagues tackle the persistent challenge of nanosilica agglomeration during fluidization. By premixing ultrafine nanosilica with small amounts of external inert silica particles classified as Geldart Groups A and B, the authors disrupt inter-agglomerate force equilibria and achieve marked reductions in minimum fluidization velocity and fluidization hysteresis. Their region-wise analysis further reveals a vertical segregation pattern, with finer Group A particles enhancing fluidization in the upper and middle bed regions, while coarser Group B particles dominate the middle and lower zones.

The microstructural and elemental characterization of calcium silicate-based sealers (CSBS) is the focus of Contribution 4 by Mateusz Radwanski and co-authors. Through SEM, EDX, and XRD analyses, they compare four commercial CSBS against a resin-based control, demonstrating that CSBS surfaces exhibit increasing calcium content upon incubation in Hank’s balanced salt solution, thereby alkalinizing the local environment and promoting mineralization and antibacterial potential. These findings contribute to the rational selection of endodontic sealers in clinical practice.

Kai Zhou and Lili Cai (Contribution 5) introduce an atmospheric flame vapor deposition (FVD) strategy for the one-step, scalable synthesis of one-dimensional V_2_O_5_ nanorods and two-dimensional V_2_O_5_ nanoflakes on diverse substrates. By fine-tuning source and substrate temperatures, they obtain highly crystalline nanostructures within seconds, and they further show that an externally applied electric field enhances the uniformity and coverage density of 2D nanoflakes. This method significantly lowers the barrier for integrating advanced vanadium oxide nanomaterials into energy and sensing devices.

In Contribution 6, Qilong Hao and co-workers investigate the synthesis and interconversion of cinnabar (α-HgS) and metacinnabar (β-HgS) by modulating the S/HgCl_2_ molar ratio. Their systematic comparison of sulfur sources, combined with SEM, XRD, XPS, and ICP-MS analyses, enables precise phase control and reveals distinct morphological signatures for each polymorph. Importantly, they propose and validate two strategies—sulfur addition and HgCl_2_ addition—to convert black β-HgS back to red α-HgS, offering new conservation pathways for degraded historical pigments in murals and paintings.

The intersection of materials science and artificial intelligence is explored in Contribution 7 by Gaoyang Xiong and colleagues, who employ machine learning (ML) to optimize the magnetron sputtering preparation of thermochromic VO_2_(M) films for smart-window applications. Among four algorithms tested, the extreme gradient boosting (XGB) model achieves the highest prediction accuracy (88.52%), and SHAP-based feature importance analysis identifies substrate temperature as the most critical process parameter. Experimental validation confirms that ML-guided optimization can substantially reduce resource wastage and accelerate the development of phase-pure VO_2_ coatings.

Shaowei Wang and co-authors (Contribution 8) present a computational study on the flow behavior of nanoparticle agglomerates in fluidized beds. By incorporating porous-structure-based drag laws—particularly the drag law for fractal porous spheres—into an Eulerian–Eulerian two-fluid model, they demonstrate that accounting for the internal pore structure of agglomerates yields significantly improved predictions of minimum fluidization velocity, bubbling velocity, bed expansion, and agglomerate dispersion. Their work underscores the necessity of moving beyond solid-sphere approximations when modeling nanoparticle fluidization systems.

In Contribution 9, Boris B. Tikhonov and colleagues develop magnetically recoverable biocatalysts composed of magnetite nanoparticles coated with an ultra-thin chitosan layer (≈0.9 nm) and covalently functionalized with glucose oxidase (GOx). The optimized biocatalyst retains 100% relative catalytic activity for the oxidation of D-glucose to D-gluconic acid and can be repeatedly recovered via magnetic separation. This benign, efficient platform illustrates the potential of nanostructured supports in pharmaceutical biocatalysis.

Jiaxuan Shi and co-workers (Contribution 10) report the design of a silica-based adsorbent, NTAamide(C8)/SiO_2_-P, for the efficient and selective removal of palladium (II) from simulated high-level liquid waste (HLLW). The adsorbent exhibits a distribution coefficient of 1848 mL/g in 0.2 M HNO_3_, with separation factors exceeding 77.8 against competing fission-product ions. The adsorption process is spontaneous, endothermic, and rapid, and the material demonstrates excellent reusability, highlighting its promise for nuclear waste remediation.

Finally, Xingliang Shen and Meiwen Cao (Contribution 11) provide a comprehensive review of bicontinuous interfacially jammed emulsion gels (Bijels). They summarize recent progress in Bijel preparation methods, structural control strategies, and the utilization of Bijels as templates for fabricating porous materials with tailored architectures. The review also outlines emerging directions and applications, bridging soft-matter physics with advanced materials engineering.

## 3. Conclusions

In conclusion, the third edition of this Special Issue, “Nanomaterials for Chemical Engineering”, presents a rich tapestry of research that advances the synthesis, characterization, modeling, and application of functional nanomaterials. The collected contributions underscore the critical role of nanoscale engineering in addressing challenges across energy, environment, healthcare, and manufacturing sectors. From metal-matrix nanocomposites and superhydrophilic coatings to machine learning-guided material design and magnetic biocatalysts, this Special Issue exemplifies the vibrant interdisciplinary spirit of modern chemical engineering. I sincerely hope that the findings and insights presented herein will inspire further innovation and foster new collaborations among scientists and engineers worldwide.
